# Recognition of a Critical Functional Domain and Improved *PHOX2B* Missense Variant Interpretation by Utilization of In Silico Prediction Tools

**DOI:** 10.1155/humu/8001520

**Published:** 2026-03-08

**Authors:** Andy Drackley, Andrew D. Skol, Casey M. Rand, Debra E. Weese-Mayer, Kai Lee Yap

**Affiliations:** ^1^ Department of Pathology & Laboratory Medicine, Molecular Diagnostics Laboratory, Ann & Robert H. Lurie Children’s Hospital of Chicago, Chicago, Illinois, USA, luriechildrens.org; ^2^ Edwards Family Division of Genetics and Rare Diseases, Ann & Robert H. Lurie Children’s Hospital of Chicago, Chicago, Illinois, USA, luriechildrens.org; ^3^ Department of Pediatrics, Northwestern University Feinberg School of Medicine, Chicago, Illinois, USA, northwestern.edu; ^4^ Stanley Manne Children’s Research Institute, Chicago, Illinois, USA; ^5^ Department of Pediatrics, Division of Autonomic Medicine, Center for Autonomic Medicine in Pediatrics (CAMP), Ann & Robert H. Lurie Children’s Hospital of Chicago, Chicago, Illinois, USA, luriechildrens.org; ^6^ Department of Pathology, Northwestern University Feinberg School of Medicine, Chicago, Illinois, USA, northwestern.edu

**Keywords:** AlphaMissense, BayesDel, CADD, CCHS, congenital central hypoventilation syndrome, homeodomain, in silico predictions, *PHOX2B*, REVEL, variant interpretation

## Abstract

Pathogenic heterozygous variants in *PHOX2B* are associated with congenital central hypoventilation syndrome (CCHS), which is characterized by autonomic nervous system dysregulation severely affecting respiratory control. The interpretation of *PHOX2B* missense variation is challenging due to their rarity and the lack of available functional evidence. Consequently, most *PHOX2B* missense variants are classified as variants of uncertain significance (VUSs), complicating the timely diagnosis and clinical management of the condition. To generate an improved model for assessments of *PHOX2B* missense variants, a methodology was derived to evaluate all *PHOX2B* missense variants in the literature and public/private databases according to a consensus classification framework and assigned pathogenicity classifications. Pathogenicity prediction scores from the in silico prediction tools CADD, REVEL, BayesDel, and AlphaMissense were obtained for all variants. A weighted logistic regression in a multiple imputation framework was performed to assess the strength of evidence supporting application of ACMG/AMP guidelines′ PP3/BP4 criteria. CADD, REVEL, and BayesDel meet the predictive strengths for PP3/BP4 recommended by the Clinical Genome Resource (ClinGen). Based on their areas under the curve and low proportions of variants with indeterminate pathogenicity predictions, BayesDel and REVEL were the strongest predictive tools and should be utilized for routine *PHOX2B* missense variant assessment with this study′s calculated score thresholds for PP3/BP4 strength levels. Furthermore, the positional distribution of pathogenic and benign variants was analyzed to assess potential hotspots or critical functional domains in *PHOX2B*, and pathogenic variants were found to cluster in the homeodomain. The enrichment of pathogenic variation was substantiated by the prediction tools, supporting the use of the PM1 criterion for variants in the homeodomain. This calibration of existing computational prediction tools for *PHOX2B* missense variant classification and recognition of the homeodomain variants will enable fewer VUS classifications in favor of conclusive results, aiding in these individuals′ care.

## 1. Introduction

Congenital central hypoventilation syndrome (CCHS, MIM #209880) is a rare autonomic disorder characterized primarily by alveolar hypoventilation, abnormal to absent control of breathing, and autonomic nervous system dysregulation [1]. Heterozygous pathogenic variation involving the highly conserved paired‐like homeobox 2B gene (*PHOX2B*, MIM #603851) is the predominant known genetic etiology of CCHS [2]. *PHOX2B* pathogenic variants are disease‐defining in the CCHS phenotype, including additional anomalies such as cardiac arrhythmias, diminished pupillary response to light, and abnormalities associated with maldevelopment of neural crest‐derived structures including Hirschsprung disease or severe constipation, neural crest tumors, and esophageal dysmotility [1, 3].

Identification of a *PHOX2B* pathogenic variant is an essential component of the CCHS diagnostic criteria, and therefore, the ability to adequately evaluate the clinical significance of identified variants is critical for the diagnosis and management of this patient population [1]. The predominant and best characterized type of disease‐causing variants is polyalanine repeat mutations (PARMs), expansions of the C‐terminal polyalanine tract [2]. The nonpolyalanine repeat mutations (NPARMs) are far less common, comprising approximately 10% of CCHS cases [4]. Pathogenicity and genotype–phenotype correlations are well characterized for both PARMs and the truncating and frameshift NPARMs [4–7]. Accurate interpretation of the well‐characterized *PHOX2B* genotypes has enabled disease prognostication and prediction of the associated phenotypic features, improving and personalizing management and disease surveillance of individuals with CCHS.

Despite these advances, there is a striking lack of information to support interpretation of *PHOX2B* missense variants due to the unpredictable impact of missense variants on gene function. Assessment of *PHOX2B* missense variants′ clinical significance remains challenging due to their rarity, the scarcity of relevant clinical and experimental functional data, and the fact that a significant proportion of variants are unique to a single individual or family [4]. Consequently, the vast majority of detected missense variants are classified as variants of uncertain significance (VUSs) due to insufficient evidence of pathogenicity [8, 9], leading to delays in clinical providers diagnosing CCHS and introducing appropriate management, despite a clinical phenotype consistent with CCHS.

Numerous computational pathogenicity prediction algorithms, which use structural and/or biochemical properties of the reference and alternative amino acids, the degree of evolutionary conservation at the nucleotide and amino acid level, among other components, have been developed to aid in the interpretation of missense variants. Ensemble or metaprediction tools that combine several algorithms to produce a single numerical summary have been shown to be more effective at predicting variant pathogenicity compared to a single algorithm or ad hoc combinations of individual algorithms; however, these tools are not equally effective predictors for all genes [10, 11]. Impressively, and consequentially, Pejaver et al. found that several prediction tools are sufficiently accurate to increase the strength of evidence awarded to the PP3/BP4 criteria (*in silico algorithms support a pathogenic or benign impact*, respectively) for some variants from the “supporting” level assigned in the original ACMG/AMP guidelines (e.g., from “supporting” to “moderate” or “strong”) [8, 11]. However, Pejaver et al. also endorsed completion of similar evaluations to assess and calibrate the different prediction tools for individual genes of interest.

This study′s goal was to improve the diagnostic laboratories′ ability to accurately classify *PHOX2B* missense variants and thus aid in the clinical management of individuals potentially affected with CCHS. With limited clinical and experimental evidence for evaluation of these variants, this study was aimed at verifying appropriate computational pathogenicity prediction tools and calibrating *PHOX2B-*specific score thresholds for these tools.

## 2. Material and Methods

### 2.1. Variant Search and Curation of the Dataset

Queries of public databases (December 2023) for all reported *PHOX2B* missense variants were performed including ClinVar [9], *PHOX2B* Leiden Open Variation Database [12], DECIPHER [13], the biomedical literature, and the large patient database managed by D.E.W‐M. and C.M.R. All available clinical information (i.e., phenotype, segregation data) was obtained for each variant. Variants in gnomAD v4.1.0 [14], annotated as coding on the MANE Select [15] transcript (NM_003924.4), and their GroupMax filtering allele frequency (FAF) data were also obtained. Amino acid substitutions caused by deletion–insertions (delins) resulting in amino acid substitutions that are impossible via a single nucleotide variant (SNV), and thus do not have precalculated pathogenicity prediction scores, were excluded from analysis (e.g., c.679_680delinsTT:p.Ala227Leu); delins variants resulting in amino acid substitutions also possible via SNV (e.g., c.432_433delinsTC:p.Trp145Arg compared to c.433T>A: p.Trp145Arg) and thus possessing precalculated scores were retained as one variant. Different SNVs resulting in identical amino acid substitutions (e.g., c.564G>T: p.Lys188Asn and c.564G>C: p.Lys188Asn), without differences in predicted splicing impacts, were retained as one variant.

### 2.2. Assigning Variant Pathogenicity

#### 2.2.1. Establishing Variant Consensus Classifications

**Figure 1:**
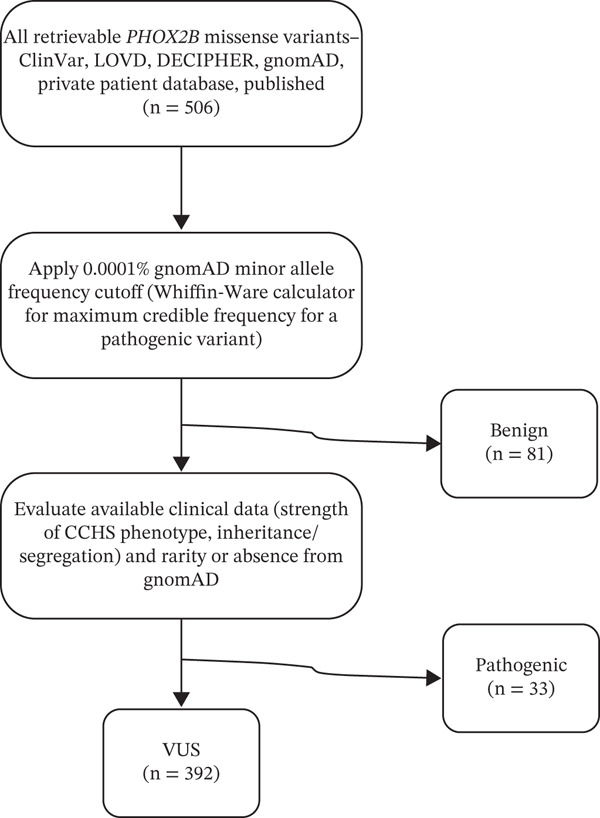
Summary of the process of obtaining the expert consensus benign, pathogenic, and VUS classification variant groups.

Nearly all *PHOX2B* missense variants have insufficient evidence to reach a (likely) pathogenic classification according to the ACMG/AMP guidelines as written due to insufficient literature support (both clinical and experimental‐based functional studies) specific to a unique variant. To create truth sets and enable critical evaluation of the performance of in silico variant effect predictive algorithms, a framework was developed for consensus classifications of variants′ presumed clinical significance; these classifications were determined by a group of clinicians and laboratorians with considerable experience in *PHOX2B* and CCHS. This framework, separate from but heavily influenced by the ACMG/AMP guidelines, is similar to a scheme by Tamana et al. for assessing variants in hemoglobinopathy genes in which, like *PHOX2B*, a huge proportion of variants are unique to a single family [16]. A visual representation of this is shown in Figure 1.

#### 2.2.2. Benign Variants

To define variants as benign, it was necessary to determine the maximum tolerated population allele frequency for *PHOX2B*/CCHS [17]. The following factors used in this calculation were conservatively set to minimize the risk of inappropriately misclassifying variants as benign: a CCHS prevalence of 1/100,000, more common than the highest current estimate of its prevalence [18]; a penetrance of 0.40 (40%) to allow for incomplete penetrance and variable expressivity or somatic mosaicism resulting in subclinical disease [5, 19]; a genetic heterogeneity of 0.75 to account for the CCHS cases without diagnostic *PHOX2B* variants identified; and an allelic heterogeneity of 0.10, representing the approximate proportion of CCHS cases caused by NPARMs and thus a highly conservative estimate for individual missense variants. This yielded a maximum tolerated allele frequency of 0.0001% (0.000001). All variants with a gnomAD v4.1.0 GroupMax FAF (joint exome and genome datasets) that exceeded the disease‐specific frequency threshold of 0.0001% were classified as benign [20].

#### 2.2.3. Pathogenic Variants

Variants were classified by the authors as pathogenic through evaluation of the following factors: The variant is present in one or more affected probands, with more weight given if the proband(s) are documented to have a formal CCHS diagnosis or a multisystemic *PHOX2B*‐related phenotype (i.e., CCHS, Hirschsprung disease, and neural crest tumors); family testing demonstrates cosegregation of the variant with *PHOX2B*‐related disease in family members, or the variant is found to be de novo in the absence of a relevant family history; the variant is extremely rare or absent in gnomAD v4.1.0; and experimental evidence is supportive of a deleterious impact of the variant.

#### 2.2.4. VUSs

Variants that did not meet the above criteria for benign or pathogenic, had insufficient or conflicting evidence, or consensus among evaluators could not be reached were classified as VUSs.

### 2.3. Computational Prediction Tools

CADD [21], REVEL [22], BayesDel [23] (“noAF,” without incorporation of population allele frequency data to avoid double‐counting of this evidence), and AlphaMissense [24] scores were obtained for all variants using their official download sites. They have all been demonstrated in large‐scale evaluations to be high performing predictors, with REVEL, BayesDel, and AlphaMissense shown to be particularly effective [10, 11, 25]. Importantly, these tools are also free to use for clinical application, have precomputed scores for all possible single nucleotide variant missense variants, and are easily integrated bioinformatically, all important considerations for clinical diagnostic workflows. Other publicly available tools with high‐level predictive performance such as MutPred2 [26] and EVE [27] were considered for assessment in this study but ultimately did not satisfy this group′s specific needs at that time.

### 2.4. Statistical Analyses

#### 2.4.1. Identifying Prediction Tool Score Thresholds

The method proposed by Pejaver et al. uses an empirical approach to estimate the posterior probability of pathogenicity and benignity conditional on prediction score (*s*) (e.g., CADD Phred value) to set thresholds for the strength of evidence that a variant is pathogenic or benign [11]. These thresholds are based on finding the values of *s* at which the estimated “positive likelihood ratio” (lr^+^) achieves a set of predetermined values (posterior probabilities of pathogenicity [or benignity] of 0.98 [very strong], 0.61 [strong], 0.21 [moderate], and 0.10 [supporting]) (see Table 1 of Pejaver et al.). Identifying empirical estimates of these thresholds, as Pejaver et al. did, protects against biases that can be introduced through parametric modeling. However, in this study, the focus is a single gene and disease (*PHOX2B* and CCHS) and variant type (missense), which greatly limits the number of true *pathogenic* and *benign* variants that can be used to estimate pathogenic/benign thresholds. Using empirical estimation with such a small number of variants would result in extremely conservative boundaries. Thus, as an alternative, a weighted logistic regression in a multiple imputation framework was used. This model is attractive in that logistic regression innately models odds ratios, an important component of lr^+^, and multiple imputation allows us to extract additional information from the VUSs (a majority of the variants) to improve the accuracy and precision of the parameter estimates. The method takes place in four steps. First, an initial weighted logistic regression using only variants with known pathogenicity/benignity is used to create a model that predicts pathogenicity as a function of *s*. Weights are used to account for the excessive sampling of pathogenic variants in the study dataset. Weights for pathogenic and benign variants were set to *P*_Path_/*P*_Path_samp_ and (1 − *P*_Path_)/(1 − *P*_Path_samp_), respectively, where *P*_Path_ and *P*_Path_samp_ are the estimated proportion of pathogenic missense variants in the population and the study sample (omitting unclassified and synonymous variants). Second, 25 imputation datasets were generated by assigning pathogenic/benign status to VUSs using the probability estimates from the model above. Third, for each imputed dataset, weighted logistic regression was performed using all variants where weight = 1 for the variants with known pathogenicity/benignity (the variants used in the initial model), and 2 *x*|0.5 − *p*_*i*,*p* *a* *t* *h*_| for the *i* ^th^ VUS. Finally, Rubin′s rule was used to incorporate the results from the multiple analyses into the parameter estimates and their covariance matrix [28]. Using these results, the odds of pathogenicity conditional on prediction score and the odds of a variant being pathogenic could be estimated; both of which are used to calculate the lr^+^. Specifically,

lr+=psv is pathogenicpsv is benign=pv is pathogenicspv is benignspv is pathogenicpv is benign=eβ0∧+β1∧s×PPath1−PPath

**Table 1:** Mean pathogenicity prediction scores and areas under the receiver operating curve for expert consensus benign and pathogenic variants in *PHOX2B.*

Prediction tool	Benign variants	Pathogenic variants	*p* value (difference)	AUC
CADD	19.00	29.00	2.0 × 10^−21^	0.963
REVEL	0.36	0.90	3.2 × 10^−30^	0.982
BayesDel	−0.14	0.49	2.9 × 10^−29^	0.992
AlphaMissense	0.30	0.96	4.7 × 10^−29^	0.961

As suggested by Pejaver et al., the 95% lower bound of the estimate of the odds was used in lieu of the odds directly. This lr^+^ lower bound was used to identify the prediction score (*s*) satisfying the pathogenicity class values in Table 1 of Pejaver et al. is

lrLB+=eβ0∧+β1∧s+1.64σ∧β02+s2σ∧β12+2s∙covσ∧β02,σ∧β12×PPath1−PPath.

The same formula is used to determine benign prediction score thresholds, except that the odds are inverted and the upper confidence bound of the odds, rather than lower, is used (e.g., change the sign in front of 1.64). An estimate of 0.044 × (1 + P_missense_) for *P*_Path_ was used based on the Pejaver et al. estimate of *P*_Path_ and adjusted for the exclusion of nonmissense variants. One‐dimensional root finding was performed using the *uniroot* function in R [29] to find the prediction score (*s*) that solved for $l{r^{+}}_{LB}$ for the likelihood ratios (LRs) given in Table 1 of Pejaver et al. [11]

#### 2.4.2. Exploring the Predictive Value of Pathogenicity Predictions

The area under the curve (AUC) of the receiver operator curve (ROC) was calculated using 80% of the data as a training set. The multiple imputation method and the modest number of variants available to build testing and training sets were accounted for by taking 200 random training and testing samples of variants. Each sampled training dataset (and thus test dataset) was made up of a random variant set with the same proportion of pathogenic/benign/uncertain variants as in the full dataset (the remaining variants made up the test set [minus the uncertain]). Each sampled training set was used to produce a predictive model as described in the previous section except that imputation and subsequent parameter estimation were performed only once (instead of 25 times). This predictive model was then used to estimate the probability of pathogenicity for each variant in the testing set, which served as input to calculate AUC using the *ROCR* package in R. The mean AUC was taken over the 200 estimates.

An ROC plot was generated using a single imputed set of variants. This ROC curves is a good estimate of the true ROC curve given the small variability in AUC values observed across imputations.

The prediction tool score thresholds described earlier were used to calculate the number of variants in the consensus VUS set that could be assigned different PP3/BP4 strength levels.

## 3. Results

The dataset comprised 506 missense variants. Through comprehensive assessment by a group of experts, 81 variants (16.0%) were classified as benign, 33 variants (7.7%) were classified as pathogenic, and 392 variants (65.2%) were classified as VUS (Figure 1 and Supporting information 1: Table S1). Each annotation′s average difference between scores in pathogenic and benign variants differed significantly (Table 1). The AUCs for CADD, REVEL, BayesDel, and AlphaMissense (Figure 1) were 0.963, 0.982, 0.9992, and 0.961, respectively.

Pejaver et al. suggest using the posterior odds of pathogenicity (or benignity) to categorize a variant′s strength of evidence for pathogenicity (or benignity) [11]. Logistic regression is well parameterized to determine the strength levels, as its regression parameter represents the change in log odds of a variant′s pathogenicity resulting from a one unit increase in prediction score. The change in the log odds of pathogenicity per prediction score unit is given in Table 2. The prediction score thresholds for all PP3 and BP4 strength levels are provided in Table 3.

**Table 2:** Estimated change in the log (OR) as a function of pathogenicity prediction tool scores.

Prediction tool	Coef	SE (Coef)	*p* value	AUC
CADD	1.10	0.17	1.1 × 10^−6^	0.963
REVEL	15.44	2.45	1.6 × 10^−6^	0.982
BayesDel	15.82	2.71	5.0 × 10^−6^	0.992
AlphaMissense	11.31	2.08	1.4 × 10^−5^	0.961

Abbreviations: Coef, regression coefficient; SE, standard error of regression coefficient.

**Table 3:** Estimated score threshold intervals for the four pathogenicity prediction tools evaluated in this study as they relate to the different pathogenic and benign strength levels.

Prediction tool	Benign (BP4)				Pathogenic (PP3)			
	Very strong	Strong	Moderate	Supporting	Supporting	Moderate	Strong	Very strong
CADD	≤ 16.17	(16.17, 20.41]	(20.41, 22.51]	(22.51, 23.54]	[26.49, 27.33)	[27.33, 29.24)	≥ 29.24	—
REVEL	—	≤ 0.26	(0.26, 0.41]	(0.41, 0.48]	[0.71, 0.77)	[0.77, 0.90)	≥ 0.90	—
BayesDel	≤ −0.52	(−0.52, −0.22]	(−0.22, −0.07]	(−0.07, 0.01]	[0.25, 0.30)	[0.30, 0.43)	≥ 0.43	—
AlphaMissense	—	≤ 0.15	(0.15, 0.37]	(0.37, 0.48]	[0.81, 0.88)	≥ 0.88	—	—

*Note:* “—”: the given tool did not meet the posterior probability (likelihood ratio) threshold. Parentheses represent exclusion of the end value; brackets represent inclusion of the end value.

**Figure 2:**
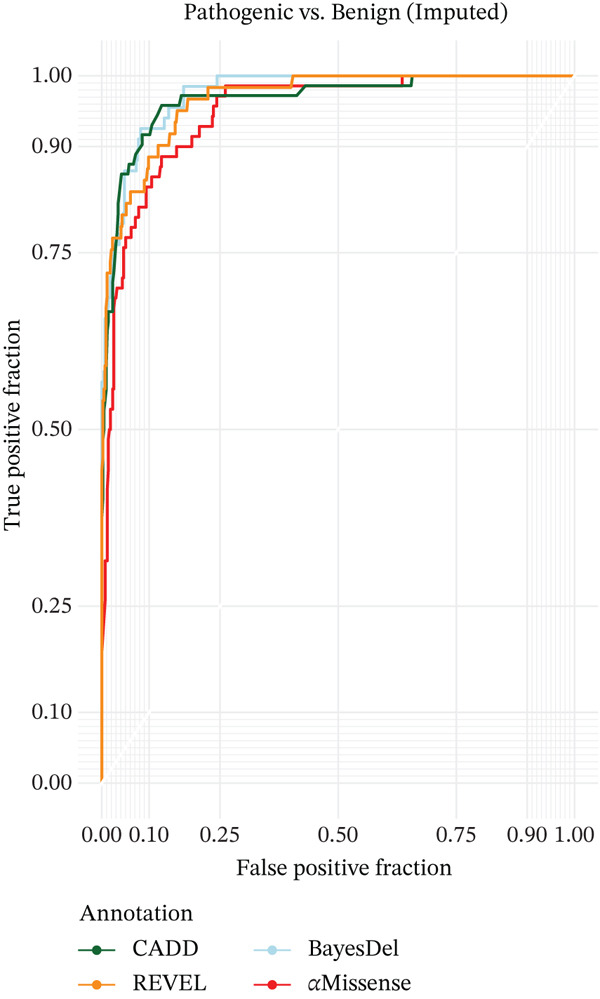
ROC curves showing the ability of the four pathogenicity prediction tools to differentiate between consensus benign and pathogenic *PHOX2B* variants (based on all variants from an imputed dataset).

The large AUC values, highly significant regression parameters (Table 2), and level of separation between consensus pathogenic and benign variants based on prediction scores (Figure 2) all point to these prediction tools performing very well.

After determining the strength thresholds, each variant in the consensus VUS set was assigned a PP3/BP4 evidence strength. Table 4 shows the number of VUSs falling into different strength levels by each of the tools. In general, classifications were very similar across prediction tools (Supporting information 2: Figure S1 and Supporting information 1: Table S1). Of the 392 VUSs, 22 had the same prediction strength across all four tools; 185 shared a prediction strength across three tools (for 135 of these variants, the disagreeing tool′s prediction strength was one level away from consensus [e.g., supporting vs. moderate]). Even among the remaining 165 variants that shared only a single strength level across tools, most (*n* = 124) had strength levels across adjacent levels. There was, however, variation across tools in the proportion of consensus VUSs predicted to be pathogenic at any strength: CADD predicted 17.9% (70/392) to be pathogenic, REVEL 12.8% (50/392), BayesDel 11.7% (46/392), and AlphaMissense 26.3% (103/392).

**Table 4:** Number of consensus VUSs (*n* = 392) predicted pathogenic or benign at different strengths.

Prediction tool	Predicted benign (BP4*_strength)*				Indeterminate	Predicted pathogenic (PP3*_strength)*			
	Very strong	Strong	Moderate	Supporting		Supporting	Moderate	Strong	Very strong
CADD	79	28	51	63	101	23	34	13	—
REVEL	—	67	131	50	94	16	22	12	—
BayesDel	1	91	132	51	71	12	21	13	—
AlphaMissense	—	104	96	28	61	22	81	—	—

### 3.1. Clustering of Pathogenic Variants in the Homeodomain

**Figure 4:**
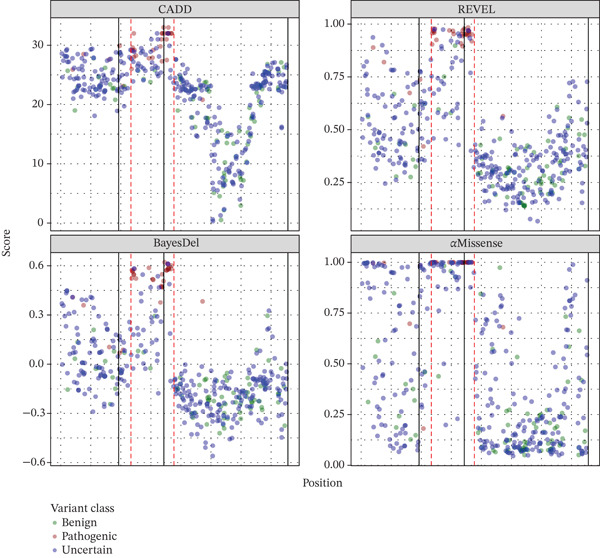
Distribution of pathogenicity prediction scores for all variants in this study. The black line denotes exon boundaries (Exon 1 on the left). The red dotted lines demarcate the homeodomain encoded by Codons 98–157.

Notably, 27/33 (81.8%) of the consensus pathogenic variants in this study were at positions between Arg99 and Arg154 in *PHOX2B* Figure 4. Codons 98–157 are responsible for encoding the PHOX2B homeodomain [30]. Examination of the distribution of the consensus benign variants demonstrated that only 4.9% (4/81) are in the homeodomain (p.Ala108Thr, p.Arg115Lys, p.Ile125Val, and p.Leu131Val). Given the concentration of consensus pathogenic variants and the paucity of consensus benign variants in the homeodomain, the four prediction tools were then used to determine whether the prediction tools support that this important protein domain may have a significant enrichment of pathogenic variation. Each tool has a significantly higher mean prediction score (*p* < 10^−8^) for all unique theoretical missense variants at positions in the homeodomain compared to the remaining theoretical variants in the rest of *PHOX2B* (Table 5).

**Table 5:** Average pathogenicity prediction scores for all unique theoretical missense variants within the homeodomain compared to those for the rest of *PHOX2B.*

Region	Prediction tool			
	CADD	REVEL	BayesDel	AlphaMissense
Homeodomain (Codons 98–157)	29.89	0.823	0.341	0.965
Nonhomeodomain (Codons 2–97, 158–314)	23.85	0.433	−0.070	0.465

Each expert consensus VUS (*n* = 392) was reinterpreted and classified according to the ACMG/AMP guidelines, incorporating this study′s recommendations for the use of PM1, PP3, and BP4 (Supporting information 1: Table S1). Of the consensus VUSs, 219 (55.9%) variants were classified as *likely benign*, four (1%) as *likely pathogenic*, and 169 (43.1%) remained as VUSs.

## 4. Discussion

As the usage of the ACMG/AMP variant classification guidelines has evolved, it is increasingly inadequate to consider variant classifications as arbitrary categorization based on rule sets. The use of a LR to define overall variant “pathogenicity” on a continuum is likely necessary [31]. With respect to in silico predictions and the PP3/BP4 criteria, nuance beyond binary prediction (benign/pathogenic) would have utility, especially when little additional evidence is available. It has already been noted that trichotomization (predicted benign vs. pathogenic vs. indeterminate) is preferred to dichotomization (predicted benign vs. pathogenic) due to the low specificity of binary predictions and their inability to meet the requisite LR for use in the Bayesian classification scheme [11, 16, 31–33]. The method used here employs a LR‐based ordinal categorization that enables us to define discrete thresholds in which the PP3/BP4 criteria are applicable at different strengths. This provides an improved resolution of in silico assessment of variant pathogenicity, while also identifying the indeterminate score ranges where in silico predictions do not increase or decrease the odds of pathogenicity.

Overall, the four computational tools all have significant predictive ability for *PHOX2B* missense variants. However, Pejaver et al. recommend that laboratories elect to use tools that can reach a “strong” level of evidence for pathogenicity and “moderate” for benignity [11]. While all four tools evaluated in this study satisfied the recommendation for benign strength predictions, AlphaMissense was the sole tool evaluated in this study that was unable to reach a “strong” strength level for pathogenic predictions and therefore should not be depended on for routine clinical assessment for *PHOX2B*. Of the three tools satisfying Pejaver et al.′s recommendations, BayesDel had far fewer “indeterminate” predictions among the study′s consensus VUS set (BayesDel: *n* = 71, CADD: *n* = 101, REVEL: *n* = 94). BayesDel also had the highest AUC (0.992) and the lowest proportion (11.7%) of predicted pathogenic variants from the VUS set, with REVEL at a similar level (12.8%) and a slightly lower AUC (0.982). The proportion of true pathogenic variants is expected to be closer to what was predicted by these two tools than by CADD (17.9%) or AlphaMissense (26.3%). The proportions of variants in the VUS set that the tools predict to be pathogenic is an important consideration as a tool should not dramatically overpredict pathogenicity (Figure 3).

Figure 3Prediction scores with pathogenic and benign evidence levels for assessed variants. (a) Consensus benign (green) and pathogenic (red) variants. (b) Consensus variants of uncertain significance. p: pathogenic; b: benign; su: supporting; m: moderate; st: strong; vs: very strong.

**Figure:**
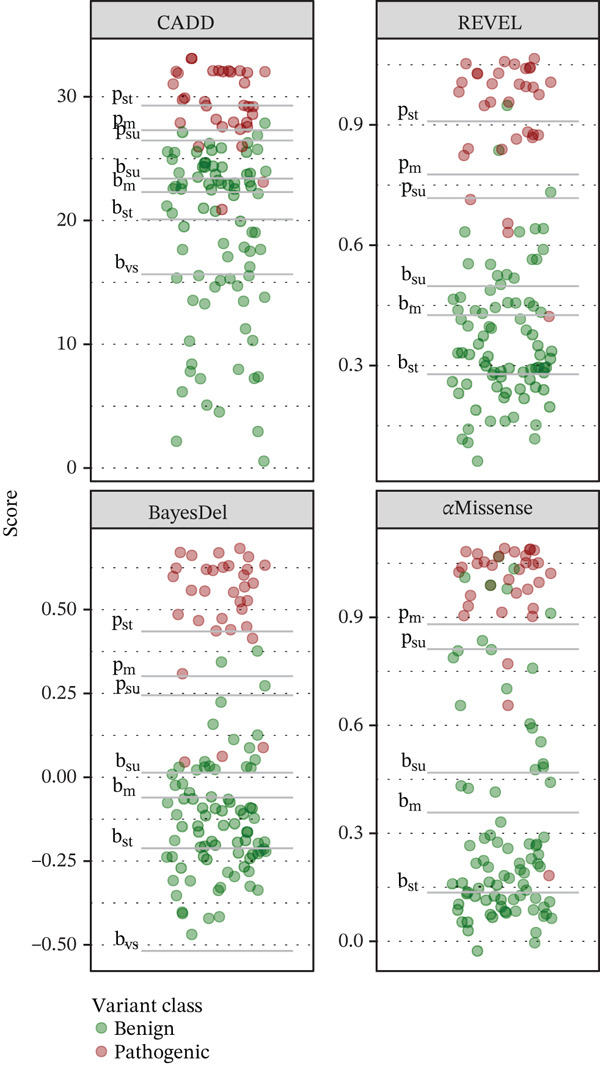
(a)

**Figure:**
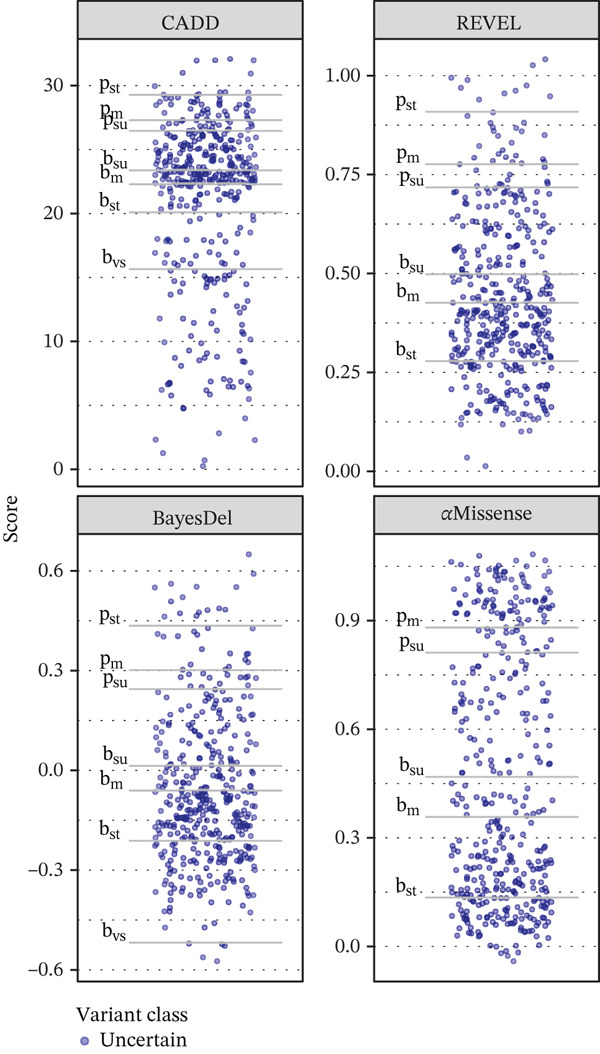
(b)

Given BayesDel′s ability to reach the requisite PP3/BP4 strength thresholds, its low rate of indeterminate predictions and highest AUC, and that the proportion of VUSs predicted to be pathogenic is closest to the presumed true burden of pathogenic variants in that cohort, BayesDel is likely the most appropriate prediction tool for routine *PHOX2B* clinical variant interpretation. REVEL, the overall performance of which is only slightly behind BayesDel, should be utilized if implementation of BayesDel scores is impossible or impractical. Both Tian et al. and Pejaver et al. found that BayesDel and REVEL outperform other tools in their large‐scale evaluations, and this study′s results support their findings for this much narrower single gene use [10, 11]. CADD, though not quite as strong a performer for *PHOX2B* in this study, is still adequate for routine use.

Additional research is necessary to determine the precise mechanism by which missense variation in *PHOX2B* leads to the isolated or syndromic CCHS phenotypes. Nevertheless, this study shows a clear enrichment of consensus pathogenic variation clustering within the homeodomain, encoded by Codons 98–157, and a significant depletion of benign variation (Figure 4). The elevated pathogenicity prediction scores for all possible missense variants in the homeodomain compared to the rest of the gene support the functional importance of this region. This is unsurprising given the PHOX2B homeodomain′s involvement in dimerization, DNA binding, and nuclear localization [34] and the recognition of the homeodomain′s importance in many other disease‐associated genes including *SHOX* (MIM #312865), *PAX3* (MIM #193500), *PITX2* (MIM #601542), and *ARX* (MIM #300382). As with *PHOX2B*, pathogenic missense variants have been found to cluster in the homeodomain‐encoding regions of these genes [35–39]. Missense variants affecting the PHOX2B homeodomain thus begin at a baseline increased likelihood of being pathogenic compared to variants outside of this domain, and the PM1 criterion (“*variant is located within a functionally critical domain or in a mutational hotspot*”) should therefore be considered for variants altering one of these 60 residues.

The use of the PP3/BP4 criteria at increased strengths and the availability of the PM1 criterion for some variants are helpful for reclassification of *PHOX2B* VUSs. These recommendations will be especially impactful in ruling out the low suspicion variants as possibly disease‐causing, as more than half of the consensus VUSs in this study met criteria for an ACMG/AMP *likely benign* classification when re‐evaluated using these modified criteria (Supporting information 1: Table S1). Evidently, these improvements in interpretation may have an important but modest impact on the reduction of VUSs in favor of (*likely*) *pathogenic* classifications until more clinical and experimental data are available. However, this reinterpretation analysis was performed predominantly on variants for which additional informative clinical data was not publicly available, and therefore, this study′s 1% reclassification of VUS to *likely pathogenic* is almost certainly an underestimation of the frequency with which variants could be upgraded to a diagnostic result in clinical practice. Even in instances when a variant is reported by a diagnostic laboratory as a VUS, this calibration of in silico pathogenicity predictors and recognition of the homeodomain′s importance can still help guide clinicians about the relative level of suspicion of pathogenicity. Though this will not resolve the issue surrounding the importance of identifying a (*likely*) *pathogenic PHOX2B* variant in the formal establishment of CCHS diagnoses in all individuals with missense variants, an improved clinician understanding of the suspicion of certain VUSs can still play a key role in informing medical management to expedite life‐saving management, genetic counseling, and expanded genetic testing decisions.

### 4.1. Case Vignette

Following completion of this study′s analyses, an external provider requested a consultation by this study′s authors on a young individual with hypoxemia and hypercarbia who had not yet undergone sleep studies nor further evaluation for CCHS. Proband‐only exome sequencing at a reference laboratory identified the previously unreported *PHOX2B* variant c.296G>C (p.Arg99Pro), reported by the laboratory as a VUS. Reassessment of this variant using this study′s recommendations suggested the variant is better classified as likely pathogenic: It is located in the homeodomain and its BayesDel, CADD, and REVEL scores of 0.572, 30.0, and 0.972, respectively, sufficient for either PP3_strong or PM1 + PP3_moderate (limiting the combined strength of PM1 and PP3 to the equivalent of a single “strong” criterion as recommended by ClinGen [11]); it occurs at the same amino acid position as another (*likely*) *pathogenic* variant (c.295C>T [p.Arg99Trp]; PM5_supporting); and it is absent from gnomAD (PM2_supporting). An improved understanding of this variant′s presumed clinical significance was critical to this individual receiving a CCHS diagnosis with the accompanying medical management recommendations and accurate counseling of the family.

### 4.2. Limitations

It cannot be ruled out that some number of variants assessed in this study may have been previously included in the training datasets of the metapredictors′ constituent tools, risking the introduction of bias into this study. However, given the small size of this study′s dataset, any potential contribution of variants assessed in this study to the constituent tools is likely to be very modest. Given the rarity of CCHS, exclusion of variants would have a dramatic impact on the ability to assess predictive performance; for this uncommon variant type associated with a very rare condition, having a complete dataset likely outweighs the risk of bias introduced by those variants′ potential inclusion.

Pairing the clinical and prediction data with experimentally derived functional data, such as that from a multiplex assay of variant effect, or MAVE [40], would further strengthen the confidence in the established score thresholds. This type of study would be of significant value to the future interpretation of *PHOX2B* missense variants.

The consensus variant classifications made in the study were agreed upon by experts involved in the clinical and molecular diagnoses of *PHOX2B*‐related conditions. These criteria were based on, but not identical to, the ACMG/AMP guidelines; there is no *PHOX2B*‐specific standard available for variant classification, and the paucity of clinical and experimental data severely limits the ability to reach (*likely*) *pathogenic* and (*likely*) *benign* classifications with strict ACMG/AMP guidelines adherence. These data are reflective of the currently available data about *PHOX2B* missense variation, and this type of analysis, including assessment of emerging or future prediction tools, should be performed periodically as clinical and population datasets continue to grow.

Lastly, though a significant update to the ACMG/AMP guidelines for sequence variant interpretation is impending, in silico predictions and gene‐level understanding including critical functional domains will persist as critical pieces of evidence for variant classification. This study′s findings will be easily transferable and equally relevant to that new classification framework.

## 5. Conclusions

In silico pathogenicity prediction tools can distinguish between pathogenic and benign missense variants in *PHOX2B* with relatively high accuracy and support the enrichment of pathogenic variation in the encoded homeodomain. This enhanced understanding is important for improving *PHOX2B* missense variant interpretation and will increase the number of conclusive classifications of variants that would have been otherwise classified as VUS, as well as provide clarity on the relative suspicion of others. All of this will positively contribute to the management of these fragile and underserved ventilator‐dependent individuals.

## Author Contributions

Conceptualization: A.D. and K.L.Y.; methodology: A.D., A.D.S., and K.L.Y.; software: A.D.S.; validation: A.D. and A.D.S.; formal analysis: A.D.S.; investigation: A.D. and A.D.S.; resources: A.D., A.D.S., C.M.R., D.E.W‐M., and K.L.Y.; data curation: A.D., A.D.S., C.M.R., D.E.W‐M., and K.L.Y.; writing—original draft: A.D. and A.D.S.; writing—review and editing: A.D., A.D.S., C.M.R., D.E.W‐M., and K.L.Y.; supervision: K.L.Y.; project administration: A.D. A.D. and A.D.S. contributed equally.

## Funding

The study is supported by the National Institutes of Health, 10.13039/100000002 (R03TR003869), and the Chicago Community Trust Foundation PHOX2B Patent Fund.

## Disclosure

All authors read and approved the final manuscript.

## Ethics Statement

The study was approved by Ann & Robert H. Lurie Children′s Hospital of Chicago′s IRB (IRB #2013‐15273).

## Conflicts of Interest

The authors declare no conflicts of interest.

## Supporting Information

Additional supporting information can be found online in the Supporting Information section.

## Supporting information


**Supporting Information 1** Table S1: All *PHOX2B* missense variants (*n* = 506) evaluated in this study. For each variant, the in silico prediction scores from all four tools are shown and are color coded according to their corresponding strength recommendations (see color key). Column K shows the variants′ expert consensus classifications. Column L shows the augmented classifications for the VUS according to the ACMG/AMP guidelines following implementation of the study′s PM1, PP3, and BP4 recommendations (using this study′s recommended tool, BayesDel); variants that received expert consensus classifications of benign or pathogenic were not reassessed as their presumed clinical significance was already established. For variants′ gnomAD GroupMax filtering allele frequencies (FAFs), “0” implies the variant is present in gnomAD v4.1.0 but its GroupMax FAF (95% confidence) is 0.

**Supporting Information 2** Figure S1. (A) Consensus pathogenic variants. (B) Consensus benign variants. Each row in the dot matrix represents a prediction tool and pathogenicity prediction. REVL = REVEL; CADD = CADD; BAYD = BayesDel; ALPM = AlphaMissense. ∗∗∗ = pathogenic_strong; ∗∗ = pathogenic_moderate; ∗ = pathogenic_supporting; U = indeterminate; ‐ = benign_supporting; ‐ ‐ = benign_moderate; ‐ ‐ ‐ = benign_strong; ‐ ‐ ‐ ‐ = benign_verystrong. The vertical connected dots represent variants observed with prediction strength combinations observed across the four prediction tools, with the count and height of the bar above it giving the number of variants observed with this combination. The bars to the right represent the marginal frequencies of each tool‐strength classification combination.

## Data Availability

All nonpatient data generated or analyzed during this study are included in this published article and Supporting information 1: Table S1. Patient‐specific data may be available from the authors upon request. Precomputed in silico prediction scores for the four tools analyzed in this study were downloaded from their respective official sites: CADD (https://cadd.gs.washington.edu), REVEL (https://sites.google.com/site/revelgenomics), BayesDel (https://fenglab.chpc.utah.edu/BayesDel/BayesDel.html), and AlphaMissense (https://console.cloud.google.com/storage/browser/dm_alphamissense).
