# Supplementary material for: Recognition of a Critical Functional Domain and Improved PHOX2B Missense Variant Interpretation by Utilization of In Silico Prediction Tools
Source: Hum Mutat. 2026 Mar 8;2026:8001520. doi: 10.1155/humu/8001520 (PMC12968325; doi:10.1155/humu/8001520)
Supplement: Supplementary file 2 — Supporting Information 2 Figure S1. (A) Consensus pathogenic variants. (B) Consensus benign variants. Each row in the dot matrix represents a prediction tool and pathogenicity prediction. REVL = REVEL; CADD = CADD; BAYD = BayesDel; ALPM = AlphaMissense. ∗∗∗ = pathogenic_strong; ∗∗ = pathogenic_moderate; ∗ = pathogenic_supporting; U = indeterminate; ‐ = benign_supporting; ‐ ‐ = benign_moderate; ‐ ‐ ‐ = benign_strong; ‐ ‐ ‐ ‐ = benign_verystrong. The vertical connected dots represent variants observed with prediction strength combinations observed across the four prediction tools, with the count and height of the bar above it giving the number of variants observed with this combination. The bars to the right represent the marginal frequencies of each tool‐strength classification combination. [file HUMU-2026-8001520-s001.pdf]

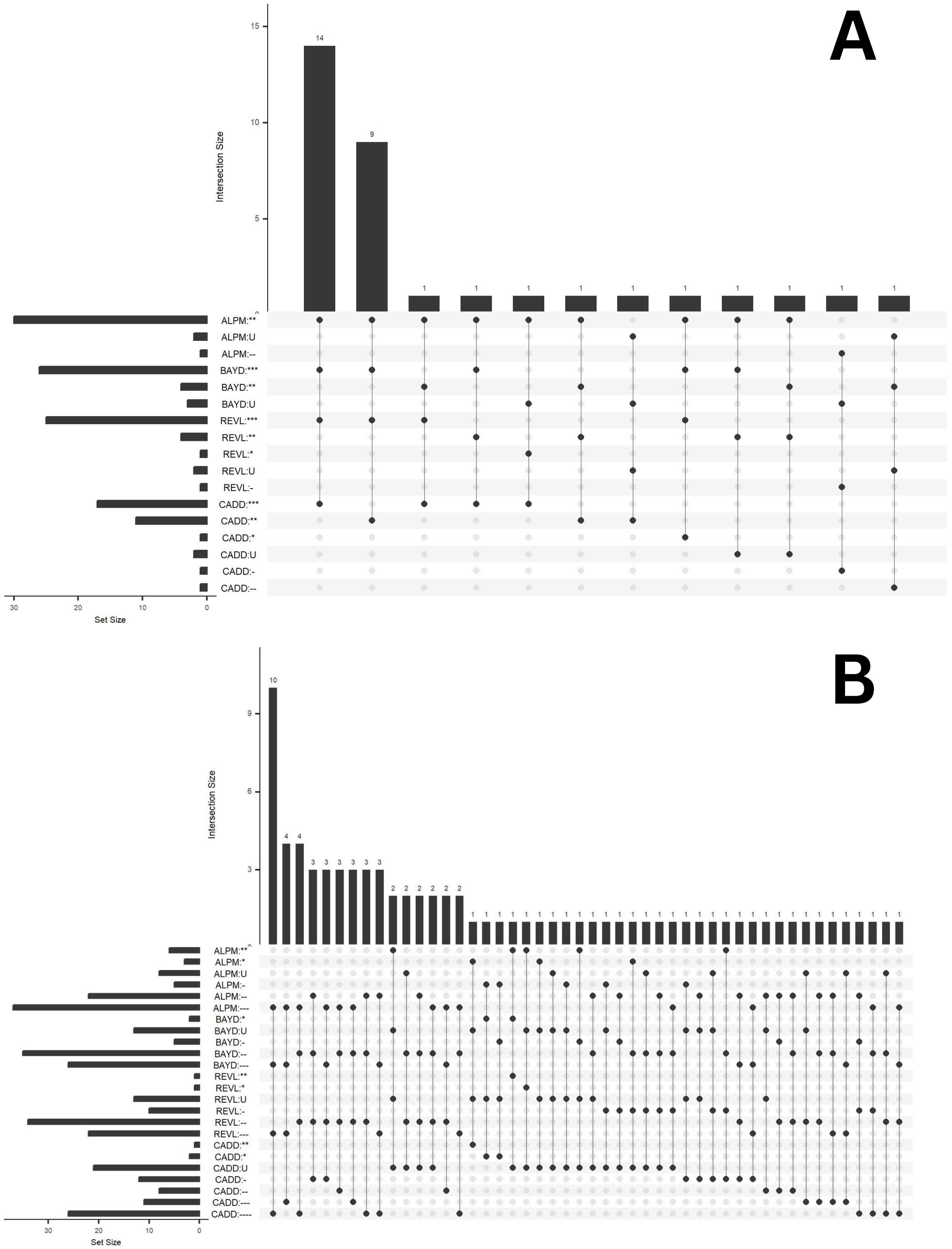

Supplementary Figure 1. (A) Consensus Pathogenic variants. (B) Consensus Benign variants. Each row in the dot matrix represents a prediction tool and pathogenicity prediction. REVL = REVEL; CADD = CADD; BAYD = BayesDel; ALPM = AlphaMissense. *** = Pathogenic_Strong; ** = Pathogenic_Moderate; * = Pathogenic_Supporting; U = Indeterminate; - = Benign_Supporting; - - = Benign_Moderate; - - - = Benign_Strong; - - - - = Benign_VeryStrong. The vertical connected dots represent variants observed with prediction strength combinations observed across the four prediction tools, with the count and height of the bar above it giving the number of variants observed with this combination. The bars to the right represent the marginal frequencies of each tool-strength classification combination.
